# A Guide to Recognizing Your Electrochemical Impedance Spectra: Revisions of the Randles Circuit in (Bio)sensing

**DOI:** 10.3390/s25196260

**Published:** 2025-10-09

**Authors:** Alexandros Lazanas, Beatriz Prieto Simón

**Affiliations:** 1 Institute of Chemical Research of Catalonia, The Barcelona Institute of Science and Technology, Av. Països Catalans 16, 43007 Tarragona, Spain; bprieto@iciq.es; 2 Institució Catalana de Recerca i Estudis Avançats (ICREA), Pg. Lluís Companys 23, 08010 Barcelona, Spain

**Keywords:** electrochemical impedance spectroscopy, Randles circuit, biosensing

## Abstract

**Highlights:**

- Suitable modifications to the Randles circuit for modern-day electrodes;
- Impact of coatings of non-biological and/or biological materials on the measured im-pedance.

**Abstract:**

Electrochemical impedance spectroscopy (EIS) is a highly versatile electrochemical technique capable of discretizing each electrochemical parameter in complex systems by employing a broad frequency spectrum. When EIS is employed in (bio)sensing applications, the electrochemical parameters are usually fitted into a relatively limited equivalent circuit model regardless of the system at hand. This work thoroughly discusses the meaning of each physical parameter in the Randles circuit, the most common equivalent circuit to model (bio)sensing systems based on EIS transduction. Additionally, it pinpoints the most suitable modifications to the Randles circuit for modern-day electrodes, where coatings of non-biological and/or biological materials can radically impact the measured impedance compared to that of unmodified electrodes. The discussion is supported by simulations that clearly exhibit the effect of each examined parameter, providing guidance for experimentalists to improve the accuracy of their work.

## 1. Introduction

Electrochemical impedance spectroscopy (EIS) is an indispensable technique employed in (bio)sensing for probing electrochemical properties associated with different biorecognition events, such as antigen–antibody interactions, protein recognition, receptor–ligand binding, and nucleic acid hybridization [1]. Because of its sensitivity to interfacial changes, EIS has been widely applied in the development of immunosensors [2]. In the case of EIS immunosensors, the binding of an antibody to its antigen at the electrode surface alters the interfacial environment, leading to measurable changes in the electrical signal [3]. The general principle in EIS biosensing is that the formation of the target–biorecognition element induces an increase in charge transfer resistance ($R_{ct}$), which directly correlates with the number of bound molecules.

**Figure 1:**
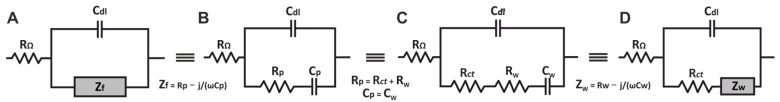
Different representations of the Randles equivalent circuit, (**A**) plain faradaic impedance, (**B**) polarization resistance and capacitance in series, (**C**) charge transfer resistance in series with Warburg resistance and capacitance, and (**D**) charge transfer resistance in series with the Warburg impedance.

Interestingly, EIS-based biosensing does not utilize the EIS discretization characteristics that are widely explored in energy-related applications. Instead, EIS-based biosensors mostly limit the information acquired by working under faradaic conditions, where a redox molecule is added to the measuring solution as a sensing probe, correlating the reversible oxidation/reduction effect with changes in the target analyte concentration. Under these conditions, a straightforward redox reaction occurs on a planar electrode (e.g., a glassy carbon electrode, GCE), characterized by a specific time constant τ, which appears as a semi-circle on the Nyquist plot, the most common EIS data representation. In this case, the impedimetric data can be fitted using a Randles equivalent circuit consisting of the ohmic resistance, the simultaneous contribution of the electrochemical double-layer capacitance and the charge transfer resistance accounting for the electron transfer between the electrode and the redox molecule, and finally the Warburg impedance, related to the mass transfer phenomena representing the “semi-infinite” diffusion of the ionic and redox species [4,5]. A representation of the Randles circuit is given in Figure 1D.

However, to meet the required analytical performance for each specific application, the majority of current EIS (bio)assays rely on surface electrode modifications with functional (bio)materials, conducting polymers, metal oxides, etc. Depending on the homogeneity and the physicochemical properties of the modified electrode surface, EIS profiles can vary greatly. Nevertheless, most published articles seem to overlook this fact by using EIS to account for electrochemical phenomena only in the vicinity of the electrode, applying the simplest model to fit data, namely the Randles circuit. This article aims to reveal the wider potential of EIS within a sensing context in a simple manner, by theoretically pinpointing key selected cases where a more complex but accurate modification of the Randles equivalent circuit can deliver a more comprehensive description of the electrochemical pattern in each assay. Such theoretical discussion is further supported by EIS simulations with varying parameters related to the emergence of surface inhomogeneity, such as the increase in the number of time constants, or the more complex mass transfer regimes in cases of coatings, and the effect of them on the different emerging capacitances in an EIS experiment.

## 2. Methodology and Key Terminology

### 2.1. Apparatus

Simulations were conducted using the “Equivalent circuit” and “simulate” routines in Iviumsoft software, v.4.12 (**Ivium Technologies** De Zaale, 11 5612 AJ, Eindhoven, The Netherlands).

### 2.2. Terminology

*Nyquist plot:*

The Nyquist plot (also known as Argand plot, Cole–Cole plot, Sluyters plot, etc.), the most common representation of EIS data, is a parametric plot of a frequency response in Cartesian coordinates, where the real part of a transfer function (in this case impedance or Z) is plotted in the X-axis (Z_real_), while the imaginary part is plotted in the Y-axis (−Z_im_). Data depiction is performed from left (high-frequency region) to right (low-frequency region) [6].

*Ohmic or uncompensated resistance:*

The ohmic resistance ($R_{\Omega }$), also referred to as uncompensated resistance [6], is a sum of the solution resistance ($R_{s}$) with the bulk resistance of the electrode ($R_{\infty }$). $R_{s}$ is an ohmic resistance attributed to the current transfer between the electrolyte solution and the electrode, achieved through ion migration [5]. Since $R_{\Omega }$ depends on both the electrolyte and the resistivity of the electrode, the ionic conductivity of the electrolyte and the resistivity of the electrode material dictate its value. Additionally, the geometry of the electrode also influences $R_{\Omega }$, with planar, spherical, and cylindrical electrodes exhibiting different values even for similar systems [6].

*Electrochemical double-layer capacitance:*

Electrochemical double-layer capacitance ($C_{dl}$) stems from the formation of two electrostatically bound layers, one formed by excess surface charge on the electrode surface and one with ions of opposite polarity. Its value plays a big role in the interfacial impedance of the electrode in tandem with the charge transfer resistance ($R_{ct}$), defining both the form of the overall impedance data and the electrocatalytic behavior.

*Constant phase element:*

As suggested above, $C_{dl}$ is the interfacial capacitance induced by the formation of a double layer close to the electrode’s surface. In an ideally planar electrode, $C_{dl}$ should remain the same throughout its surface. However, in real electrodes, there is a capacitance fluctuation across their surface, which can be attributed to numerous parameters (e.g., the roughness of the electrode surface, surface impurities, and adsorbed compounds). The term “constant phase element” stems from the fact that the phase angle of the portion of a circuit represented by such an element is AC frequency independent [7]. To this day, the reason for its presence has not been fully justified [8]. Nonetheless, this deviation from ideal $C_{dl}$ behavior has been accounted for by using a constant phase element (CPE) instead of a standard capacitor, with the inclusion of an exponent (n) which values vary from 1 (ideal capacitor) to 0 (ideal resistor), although experimental values below 0.5 should be carefully considered [5]. The formula for the impedance of the CPE is given below [8]:

(1) $$Z_{CPE}=\frac{1}{Y_{0}{\left(j\omega \right)}^{n}}$$

where $Y_{0}$, given in F s^n−1^, is the parameter containing the capacitance information [5].

*Charge transfer resistance:*

The charge transfer resistance ($R_{ct}$) is another ohmic resistance attributed to the electron transfer reaction between the electrode and an electroactive molecule present in solution or confined to the electrode surface through physical adsorption or chemical binding. The value of $R_{ct}$ depends on the kinetics of the system (heterogeneous electron transfer rate constant—$k^{0}$), and the electroactive surface area (A) of the electrode (Rct=RTn2F2ACk0). It should be noted that this relationship is derived from the Butler–Volmer equation when small overpotential values are considered (up to ca. 50 mV) [4,9]. In any case, for electrodes with an electrocatalytic surface suitable for the electrochemical reaction, then $R_{ct}$ is expected to be small and vice versa. In faradaic EIS bioassays $R_{ct}$ is more often than not the measuring parameter, either as an absolute value or as its relative change.

*Randles equivalent circuit:*

The Randles equivalent circuit is a proposed model circuit for the representation of “simple” electrochemical processes in the presence of an electroactive molecule (in solution). A first depiction of the Randles circuit is shown in Figure 1A, at high frequencies, the electrode/electrolyte resistance emerges, and the semi-circle formed from high to intermediate frequencies represents the simultaneous contribution of the capacitive ($C_{dl}$) and the faradaic impedance ($Z_{f}$) [4]. An equivalent representation of this circuit would emerge by depicting $Z_{f}$ as one resistor and one capacitor in series ($R_{p}$ and $C_{p}$) (Figure 1B) [4]. This representation is useful in systems showing the sequential effect of polarization and capacitance. A more common representation of the Randles circuit is expressed if we equalize $R_{p}$ to the sum of $R_{ct}$, accounting for the charge transfer between the electrode and the electroactive species, and $R_{w}$ (Warburg resistance). At the same time, we can equalize $C_{p}$ to the respective capacitance parameter $C_{w}$ (Warburg capacitance) (Figure 1C). Finally, the sum of $R_{w}$ with the respective reactance (−$j/\omega C_{W}$) represents the well-known Warburg impedance ($Z_{w}$) accounting for the “semi-infinite” mass transfer of the electroactive species (Figure 1D) [4,5]. In the low-frequency region, mass transfer phenomena dominate the impedimetric behavior, exhibiting a 45° straight line that represents the diffusion of the electroactive species [10]. To summarize, starting from high to low frequencies, at the highest frequency, there is $R_{\Omega }$, and the semi-circle formed from high to intermediate frequencies represents the simultaneous contribution of the $C_{dl}$ and $R_{ct}$, representing the electron transfer between the electrode and the redox molecule. In the low-frequency region, mass transfer phenomena dominate the impedimetric behavior, showing a 45° degrees straight line representing the “semi-infinite” diffusion of the electroactive species.

Most works employ the Randles circuit as a panacea for all electrochemical systems, but such a simple approach has some issues that need to be considered. First of all, biosensing assays rely on the use of biomolecules as biorecognition elements bound to the electrode surface through coupling chemistry. These biomolecules (e.g., antibodies and aptamers) are mostly dielectric in nature and thus deteriorate the charge transfer between the electrode and the electroactive species. Additionally, the influence of dielectric coatings on the degree and type of mass transfer is significant, an effect that will be further discussed. As a final remark, it should be stressed that this work does not serve as a detailed retrospective article of recent EIS biosensing works, but rather as a concise text that can help readers adjust experimental data to the appropriate equivalent circuit model. Nevertheless, for the reader who wishes to obtain a more detailed analysis of EIS biosensor publications, we suggest the following works [1,3,11,12]. The authors of [11,12] provide a meaningful discussion on the importance of selecting the correct equivalent circuit and its impact on the appropriate fitting of EI spectra.

## 3. Results

### 3.1. Semi-Infinite Mass Transfer

#### 3.1.1. Influence of Mass Transfer Time Constant

The time constant of an RC circuit is given by [5]:

(2) τ=RC

Consequently, the faradaic kinetics of an electrochemical system are assessed by their characteristic time constant ($\tau_{f}$):

(3) $$\tau_{f}=R_{ct}C_{dl}$$

$\tau_{f}$ is a measure of the rate of the faradaic kinetics of the electrochemical system, meaning smaller $\tau_{f}$ values equal faster charge transfer reactions between the electrode and the electroactive molecule (either in solution or immobilized on the electrode surface).

Conversely, the mass transfer kinetics of a Randles circuit are expressed by the Warburg element, denoting the linear semi-infinite diffusion. Warburg impedance ($Z_{W}$) refers to the semi-infinite mass transfer of electroactive molecules and ions from the bulk volume of the electrolytic solution to the electrode–electrolyte interface, where the electrochemical reaction takes place. The term “semi-infinite” implies that the distance from the bulk solution that the molecules/ions have to travel from is time-dependent (non-steady state), and the diffusion layer extends to a seemingly “infinite” distance from the bulk solution side while remaining bounded from the electrode–electrolyte interface side (thus the term “semi-infinite” instead of “infinite”). As mentioned above, $Z_{W}$ can be given as a resistor and a capacitor in series, where [4]

(4) $$R_{W}=\frac{\sigma }{\sqrt{\omega }}\;\mathrm{and}\;C_{W}=\frac{1}{\sigma \sqrt{\omega }}$$

where σ (given in Ω ${cm}^{-2}$) is the mass transfer impedance parameter [10]:

(5) $$\sigma =\frac{RT}{n^{2}F^{2}A\sqrt{2}}\left(\frac{1}{\sqrt{D_{0}}C_{0}^{*}}+\frac{1}{\sqrt{D_{R}}C_{R}^{*}}\right)$$

n is the number of electrons transferred in the electrochemical reaction, F is Faraday’s constant (96,485 C mol^−1^), R is the gas constant (8.314 J mol^−1^ K^−1^), T is the temperature (K), A is the electroactive surface area of the working electrode (cm^2^), and $C_{0}^{*}$ is the concentration of the redox species in their oxidized form, while $C_{R}^{*}$ is the concentration of the redox species in their reduced form (the asterisk refers to the concentration of the species in the bulk solution). Respectively, $D_{0}$ is the diffusion coefficient (cm^2^ s^−1^) of the redox species in their oxidized form and $D_{R}$ is the diffusion coefficient of the reduced form of the redox species. If we assume that the concentration adjacent to the electrode is the same as the one in the bulk solution and that $C_{0}^{*}=C_{R}^{*}=C$, as well as that the diffusion coefficient of the oxidized and reduced species is the same $\;D_{0}=D_{R}=D$ (this is not always the case), then we get [5]:

(6) $$\sigma =\frac{2RT}{n^{2}F^{2}A\sqrt{2}DC}$$

(7) $$Z_{W}=R_{W}-\frac{j}{\omega C_{W}}=\frac{\sigma }{\sqrt{\omega }}-j\frac{\sigma }{\sqrt{\omega }}=\frac{\sigma }{\sqrt{\omega }}\left(1-j\right)=\sigma \sqrt{\frac{2}{j\omega }}$$

The last equation can be found through the $1-j=\sqrt{\frac{2}{j}}$ conversion [13]. Now, considering that in its nature, $Z_{W}$ is a CPE with $Y_{W}$ as the equivalent admittance (given in s^1/2^ ohm^−1^ or F s^−1/2^) and n exponent value of 0.5, then [13]

(8) $$Z_{W}=\sigma \sqrt{\frac{2}{j\omega }}=\frac{1}{Y_{W}\sqrt{j\omega }}$$

(9) $$Y_{W}=\frac{1}{\sigma \sqrt{2}}$$

Hence, we can find the time constant by considering the average capacitance of the Warburg CPE, using a modified Brug’s formula [14]:

(10) $$\bar{C}=Y_{W}^{1/n}{\left(R_{ct}\right)}^{\frac{1-n}{n}}=Y_{W}^{2}R_{ct}=R_{ct}/(2\sigma^{2})$$

Then the mass transfer time constant becomes

(11) $$\tau_{d}=R_{ct}\bar{C}={R_{ct}}^{2}/(2\sigma^{2})$$

It is noteworthy that $\tau_{d}$ depends equally on σ and $R_{ct}$, meaning that the fast diffusion kinetics are achieved in tandem with fast charge transfer kinetics (small $R_{ct}$ values) and/or large σ values (small diffusion coefficient and/or concentration of redox species).

Finally, by combining Equations (3) and (11), the ratio of the mass transfer and faradaic time constants equals

(12) $$\frac{\tau_{d}}{\tau_{f}}=\frac{\left(\frac{{R_{ct}}^{2}}{2\sigma^{2}}\right)}{\left(R_{ct}C_{dl}\right)}=\frac{R_{ct}}{2\sigma^{2}C_{dl}}$$

One important consideration in impedimetric research is how the $\frac{\tau_{d}}{\tau_{f}}$ ratio can influence the data acquired from the EI spectrum. VanderNoot [15] investigated the impact of this ratio and concluded that, to gain enough information out of the spectrum, the $\frac{\tau_{d}}{\tau_{f}}$ ratio should be higher than 1. However, one aspect that has not been thoroughly investigated is which parameter plays a larger role and/or changes the spectrum more dramatically. More specifically, $C_{dl}$ is a parameter solely related to $\tau_{f}$, while $R_{ct}$ is related to both $\tau_{d}$ and $\tau_{f}$.

**Figure 2:**
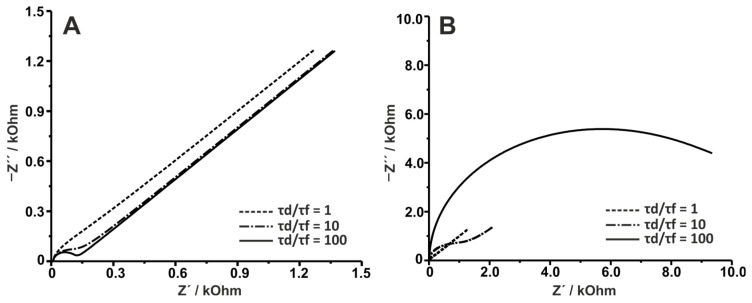
Simulated Nyquist plots of a Randles circuit with $R_{\Omega }$ = 10 Ω and $Y_{W}$ = 7.071 × 10^−4^ F s^−1/2^. (**A**) Different $\tau_{d}$/$\tau_{f}$ ratios with $R_{ct}$ = 100 Ω and (dashed line) $C_{dl}$ = 5 × 10^−5^ F, (dotdash line) $C_{dl}$ = 5 × 10^−6^ F, and (solid line) $C_{dl}$ = 5 × 10^−7^ F. (**B**) Different $\tau_{d}$/$\tau_{f}$ ratios with $C_{dl}$ = 5 × 10^−5^ F and (dashed line) $R_{ct}$ = 100 Ω, (dotdash line) $R_{ct}$ = 1000 Ω, and (solid line) $R_{ct}$ = 10,000 Ω.

To shed light on that matter, Figure 2A shows simulated Nyquist plots with $C_{dl}$ being varied in three cases, leading to $\frac{\tau_{d}}{\tau_{f}}$ ratios of 1, 10, and 100. It is clear that the higher the $\frac{\tau_{d}}{\tau_{f}}$ ratio, the higher the resolution between the kinetic part of the plot (semi-circle) and the mass transfer part of the plot (45° line). The $\frac{\tau_{d}}{\tau_{f}}$ ratio of 100 seems to provide the best separation between the two parts of the plot. Conversely, Figure 2B shows the simulated Nyquist plots with $R_{ct}$ being varied while $C_{dl}$ remains constant. In this case, increasing the $\frac{\tau_{d}}{\tau_{f}}$ ratio by increasing $R_{ct}$ seems to lead to the gradual disappearance of the semi-infinite mass transfer part of the plot. It is evident that the ratio of the two time constants is not singlehandedly the defining factor to attain the best resolution between the kinetic and the mass transfer part of the plot. Therefore, to gain good resolution, $R_{ct}\geq \frac{\sigma }{\sqrt{\omega }}$ but also $R_{ct}$ cannot be so large as to force the AC to flow directly through $C_{dl}$, then $R_{ct}\leq \frac{1}{\omega C_{dl}}$ [4].

Nyquist plots similar to the one depicted for $\frac{\tau_{d}}{\tau_{f}}=100$ in Figure 2B, are often encountered in biosensing assays where the immobilization of biorecognition elements heavily impedes the charge transfer between the electrode and the redox probe, leading to excessive $R_{ct}$ values. As an example of the information that can be extracted from changes in mass transfer and faradaic time constants, EIS analysis was used in a study aimed at comparing the efficiency of three protein immobilization methods [16]. Specifically, cytochrome c (cyt c) was attached to a gold electrode that had been previously functionalized via the self-assembly of mercaptopropionic acid (MPA). The three methods tested included: (1) electrostatic interactions between negatively charged carboxylic groups on the electrode surface and positively charged cyt c, (2) covalent binding of cyt c to the carbodiimide-activated electrode surface, and (3) interactions between Zr(IV) attached to the self-assembled monolayer and cyt c. Cyt c immobilization was confirmed by the blocking (impedimetric) effects against the redox probe added to the measuring solution. This blocking effect was used to assess the immobilization method based on the amount of cyt c attached to each electrode. Here, it is important to note that no electroactive species were used in solution, as cyt c is electroactive itself, resulting in a faradaic behavior observed as a slight deviation from the EIS complex plane plots. The modified Randles’ model used to fit EIS data included two CPEs, one corresponding to the $C_{dl}$ and another to an adsorption capacitance parameter that arises from the confined redox reaction (i.e., cyt c attached to the surface). Protein surface coverage (Γ), calculated as $\Gamma =4RTC/n^{2}F^{2}A$, using C from EIS data fittings confirmed that the Zr-based immobilization method led to the highest coverage, while the carbodiimide-based method provided the lowest yield. Interestingly, the time required to perform EIS measurements, combined with the limited stability of the electrostatic interactions, added uncertainty to the calculated Γ values. More examples with similar EIS behavior are given here [17,18].

#### 3.1.2. Semi-Infinite (Hemi)spherical Diffusion

Added to the common semi-infinite linear diffusion observed for a regular electrode, there are other EIS-relevant cases. Semi-infinite (hemi)spherical diffusion is usually associated with ultramicroelectrodes, which possess at least one critical dimension at the micrometer scale (or at the sub-micrometer scale) [19]. This changes the mass transfer region of the EIS plots when the diffusion layer becomes larger than the radius ($r_{0}$) of the electrode. Note that both conditions described herein refer to external diffusion (i.e., diffusion in solution) and not finite-length diffusion (e.g., diffusion inside a polymer film). For readers interested in that subject, few sources are suggested [5,13,20]. For semi-infinite (hemi)spherical diffusion, the Warburg element is modified with a resistor in parallel, and the modified Warburg impedance ($Z_{W}^{\prime }$) of the semi-infinite mass transfer is given by [13]:

(13) $$Z_{W}^{\prime }=\frac{\sigma^{\prime }}{\sqrt{\omega }}(1-j)=\frac{1}{Y_{W}^{\prime }\sqrt{j\omega }+\frac{1}{R_{D}}}$$

where $R_{D}$ is the diffusion resistance given as

(14) $$R_{D}=\;\frac{\sigma^{\prime }r_{0}}{\sqrt{D}}=\frac{r_{0}}{Y_{W}^{\prime }\sqrt{D}}$$

In contrast with semi-infinite linear diffusion, where $Z_{W}$ tends to infinity when ω tends to zero, for (hemi)spherical diffusion, when ω tends to zero, $Z_{W}^{\prime }$ has a real value equal to ${R_{u}+R}_{ct}+R_{D}$ (for ideally reversible systems $R_{ct}$ can be omitted).

A less-known condition where semi-infinite (hemi)spherical diffusion might present itself is the case of heterogeneous electrode surfaces (this could be either a heterogeneous coating or a pristine heterogeneous electrode surface) [21]. In this case, the electrode surface consists of active and inactive parts, depending on the dimensions of the mean sizes of the active centers and their relationship with the diffusion layer. More specifically, hemispherical diffusion will occur if the diffusion layer is larger than the mean radius of the active centers ($r_{a}$) but smaller than the mean radius of the inactive centers ($r_{i}$) [22,23]:

(15) $$r_{a}<l_{d}=\sqrt{\frac{2D}{\omega }}<\;r_{i}$$

**Figure 3:**
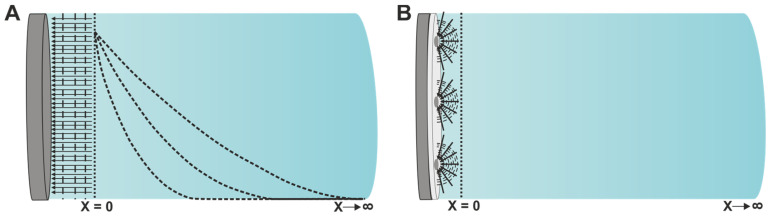
Different semi-infinite mass transfer regimes, (**A**) linear and (**B**) (hemi)spherical. X = 0 denotes the start of the diffusion layer while X →∞ denotes the bulk electrolyte solution.

Figure 3 depicts two different semi-infinite mass transfer regimes, one (A) where linear mass transfer occurs as in a regular macroelectrode, and one (B) where (hemi)spherical mass transfer takes place, in a heterogeneous electrode surface as described above. When linear diffusion occurs, as illustrated in (A), the concentration gradient is shown after the application of a potential step, denoting the diminution of electroactive species near the diffusion layer (X = 0) over long times. In contrast, the situation depicted in (B) presumably takes place after a steady-state diffusion has been achieved, which should happen for (hemi)spherical diffusion when ω is sufficient to induce the conditions of Equation (15).

**Figure 4:**
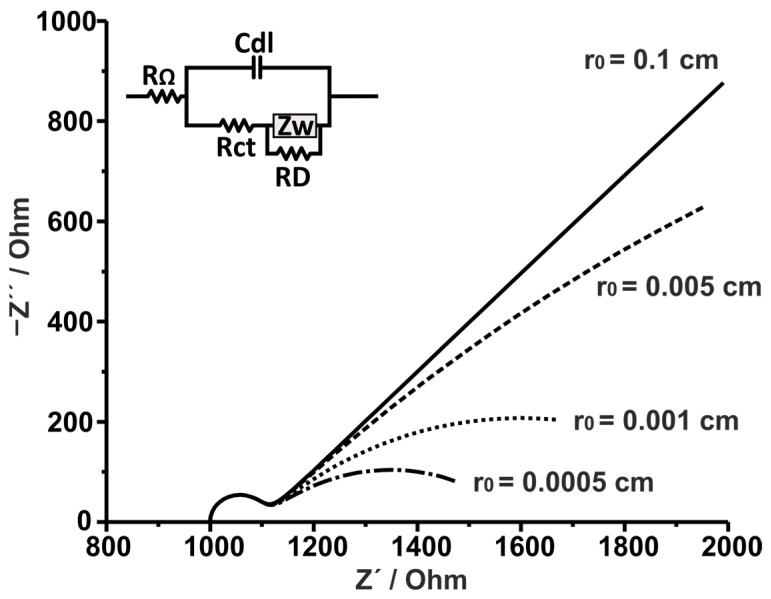
Simulated Nyquist plots of a Randles circuit under semi-infinite spherical diffusion. $R_{\Omega }$ = 1000 Ω, $R_{ct}$= 100 Ω, $C_{dl}$ = 10^−6^ F, and $Y_{W}$ = 10^−3^ F s^−1/2^; (solid line) $R_{D}$ = 100,000 Ω, (dashed line) $R_{D}$ = 5000 Ω, (dotted line) $R_{D}$ = 1000 Ω, and (dotdash line) $R_{D}$ = 500 Ω. D was set to 10^−6^ cm^2^ s^−1^.

Figure 4 illustrates simulated Nyquist plots with different mass transfer regimes based on different active radii (which can be either the radii of ultramicroelectrodes or the active areas of heterogeneous electrode surfaces). For each case, $R_{D}$ was calculated for a certain $r_{0}$ while maintaining all the other parameters constant (D was set to 10^−6^ cm^2^ s^−1^). It is evident that for a very large radius (0.1 cm), the mass transfer aligns with linear diffusion, due to the planar behavior of the electrode. As we progress to smaller radii, a curvature appears at low frequencies attributed to the (hemi)spherical diffusion effects, with 0.0005 cm producing a low-frequency second semi-circle, as expected from Equation (13). While this is a fairly common behavior in cases where macroelectrodes are used, the assessment of such spectra should be performed with special care to avoid drawing wrong conclusions. Such systems can be misapprehended to contain two characteristic faradaic time constants (RC)(RC) and can be modeled as such, inadvertently losing relevant mass transfer-related information. If the user is unsure about the physical characteristics of the system at hand, it would be more beneficial in such a case, (a) to extend the examined frequency range to lower frequencies, and/or (b) to increase the concentration of the electroactive species. This is advised because if the system is indeed (RC)(RC), then going to lower frequency ranges may reveal a previously masked diffusion region. On the other hand, if the system is driven by spherical diffusion, increasing the concentration of electroactive species could bring the system to a more linear diffusion behavior. At the same time, if the origin of the second semi-circle is inherently faradaic, increasing the concentration should just produce a smaller semi-circle.

An interesting example of hemi(spherical) diffusion in a heterogeneous electrode surface was reported by O’Connor et al. [24]. The authors electrochemically characterized the progressive growth of a bacterial biofilm on the surface of a thermally hydrocarbonized porous silicon (THC-pSi) platform. The EIS measurements were conducted in the presence of hexammineruthenium(III) chloride as the electroactive species in the electrolyte solution. At 24 h of bacterial biofilm growth, a large semi-circle was observed in the Nyquist plot, attributed to the partial blockage of charge transfer caused by the thick biofilm, which featured some pinholes. Nonetheless, at 48 h and 72 h of biofilm growth, at low frequencies, the plots deviated from the 45° slope observed for semi-infinite linear diffusion. This deviation is attributed to hemispherical diffusion, arising from an electrodisc system that combines inactive parts, corresponding to the biofilm, and active parts, represented by micro-sized gaps in the biofilm, which serve as the focus of the hemispherical diffusion field. As a result, the equivalent circuit had to be modified by adding a resistance (R_D_) in parallel to the Warburg impedance, accounting for the thinner, more homogeneous parts of the biofilm. Notably, by calculating the thickness of the diffusion layer at the lowest frequency of the spectrum (0.1 Hz), the authors estimated the mean size of the active area (micro-sized gaps) of the sensors. More examples of the influence of the (hemi)spherical diffusion can be found here [25,26,27].

### 3.2. Capacitance

**Figure 5:**
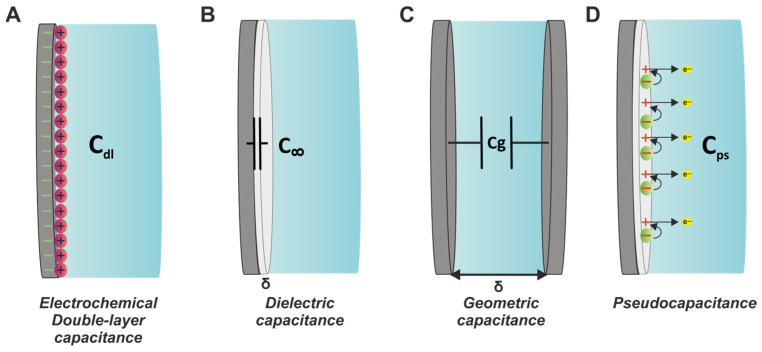
Different capacitance effects potentially present in EIS measurements (**A**) $C_{dl}$, (**B**) $C_{\infty }$, (**C**) $C_{g}$, and (**D**) $C_{ps}$.

As previously stated, the $C_{dl}$ of the electrode is an integral part of any equivalent circuit used in EIS. However, $C_{dl}$ is not the only capacitance present in impedimetric measurements, since there are other types of capacitances attributable to the electrochemical system itself. Figure 5 illustrates the four common capacitance effects relevant to EIS, which are thoroughly discussed in this work, including $C_{dl}$, dielectric capacitance ($C_{\infty }$), geometric capacitance ($C_{g}$) and pseudocapacitance ($C_{ps}$).

#### 3.2.1. Dielectric Capacitance ($C_{\infty }$)

It should be stressed that every electrode–material system in a measuring cell has a dielectric capacitance and a bulk resistance ($R_{\infty }$) in parallel with it [28]. These two elements have their own characteristic time constant, which is the dielectric relaxation time of the electrode material [28]. In fact, ohmic resistance can be considered as the sum of the solution resistance ($R_{s}$) and the $R_{\infty }$:

(16) $$R_{\Omega }=R_{s}+R_{\infty }$$

However, for most applications where metallic or carbon electrodes are used, it follows that $R_{\infty }\to$0 and thus $R_{\Omega }=R_{s}$. This is not the case when semi-conducting and/or polymeric coatings are used as electrodes or electrode modifiers. The influence of $R_{\infty }$ can be so severe as to affect measurements, especially when combined with increased dielectric capacitance.

Dielectric capacitance is often associated with dielectric coatings on the electrode surface (polymeric or other), and it can be expressed by Equation (17) [29,30]:

(17) $$C_{\infty }=\frac{\varepsilon \varepsilon_{0}}{\delta }$$

where ε stands for the dielectric constant of the coating material, $\varepsilon_{0}$ is the relative permittivity of vacuum (8.8542 × 10^−14^ F cm^−1^), and δ stands for the thickness of the coating. Equation (17) is sometimes normalized using the electroactive surface area (A), but this expression has been omitted here since the values of the simulated elements are non-normalized. Since $R_{\Omega }$ and $R_{s}$ are in parallel with $C_{\infty }$, they have their own time constant as per Equations (3) and (17) [28]:

(18) $$\tau_{\infty }={(R}_{s}+R_{\infty })C_{\infty }=R_{\Omega }C_{\infty }$$

To investigate the possibility of interference due to $C_{\infty }$, its characteristic frequency $\left(f_{c}\right)$ can be estimated using [30,31]:

(19) $$f_{c}=\frac{1}{\left(2\pi R_{\Omega }C_{\infty }\right)}$$

For regular measurements in buffer solution, $f_{c}$ is expected to be large enough as not to interfere with the EIS measurements (assuming a frequency range of 100 kHz–0.1 Hz) [30].

**Figure 6:**
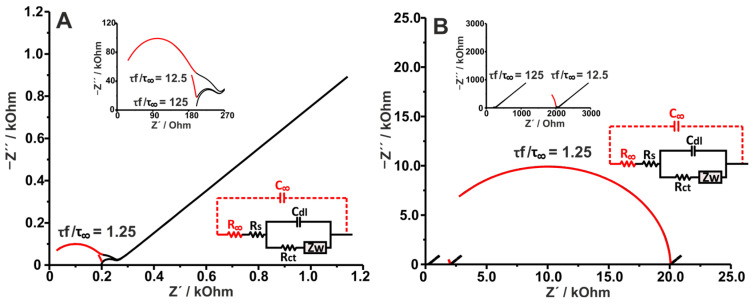
Simulated Nyquist plots of (**A**) an electrode under the capacitive effect of a dielectric coating: $R_{\Omega }$ = 100 Ω, $R_{\infty }=\;$ 100 Ω, $C_{dl}$ = 10^−4^ F, $R_{ct}$ = 50 Ω, and $Y_{W}$ = 10^−3^ F s^−1/2^; three different $C_{\infty }$ were used, (i) 2 × 10^−10^ F $\left(\frac{\tau_{f}}{\tau_{\infty }}=\;125\right)$, (ii) 2 × 10^−9^ F $\left(\frac{\tau_{f}}{\tau_{\infty }}=\;12.5\right)$, and (iii) 2 × 10^−8^ F $\left(\frac{\tau_{f}}{\tau_{\infty }}=\;1.25\right)$, and (**B**) an electrode under the resistive effect of a dielectric coating: $R_{\Omega }$ = 100 Ω, $C_{\infty }=\;$ 2 × 10^−10^ F, $C_{dl}$ = 10^−4^ F, $R_{ct}$ = 50 Ω, and $Y_{W}$ = 10^−3^ F s^−1/2^; three different $R_{\infty }$ were used, (i) 100 Ω $\left(\frac{\tau_{f}}{\tau_{\infty }}=\;125\right)$, (ii) 1900 Ω $\left(\frac{\tau_{f}}{\tau_{\infty }}=\;12.5\right)$, and (iii) 19,900 Ω $\left(\frac{\tau_{f}}{\tau_{\infty }}=\;1.25\right)$.

It is important to state that, similarly to what happens with the mass transfer time constant expressed by Equation (11), in this case, both the value of the time constant and the individual values of $R_{\infty }$ ${(R}_{s}$ is constant in this case) and $C_{\infty }$ can impact the acquired spectrum. This effect is shown in Figure 6 through the analysis of simulated Nyquist plots corresponding to different ratios of $\frac{\tau_{f}}{\tau_{\infty }}$ obtained by changing $R_{\infty }$ or $C_{\infty }$. Specifically, Figure 6A shows the impact of varying $\frac{\tau_{f}}{\tau_{\infty }}$ by increasing $C_{\infty }$. To utilize $C_{\infty }$ values that could apply to real applications, a constant ε value of 2.26 was chosen (a value that aligns with the dielectric constant expected for a polymeric coating), and the increase in $C_{\infty }$ is given as a result of the decrease in the coating’s thickness (δ). Coating thicknesses of 10 μm ($C_{\infty }$ = 2 × 10^−10^ F/$\;\frac{\tau_{f}}{\tau_{\infty }}=\;125$), 1 μm ($C_{\infty }$ = 2 × 10^−9^ F/$\;\frac{\tau_{f}}{\tau_{\infty }}=\;12.5$), and 0.1 μm ($C_{\infty }$ = 2 × 10^−8^ F/$\;\frac{\tau_{f}}{\tau_{\infty }}=\;1.25$) were employed.

Figure 6A shows that with greater $\tau_{\infty }$ values (and consequently lower $\frac{\tau_{f}}{\tau_{\infty }}$) the impact of increasing $C_{\infty }$ is severe on the EI spectra, reaching a point where the faradaic semi-circle is barely visible ($\frac{\tau_{f}}{\tau_{\infty }}=1.2$5). This proves that the dielectric time constant strongly affects the outcome of the Nyquist plot. However, it is important to remember that the change in the time constant in this case is induced by a change in $C_{\infty }$ while $R_{\infty }$ is constant. In contrast, Figure 6B shows three different time constant values, but, in this case, the change is induced by $R_{\infty }$ as follows: $R_{\infty }=$ 100 Ω/$\frac{\tau_{f}}{\tau_{\infty }}=\;125$, $R_{\infty }=$ 1900 Ω/$\frac{\tau_{f}}{\tau_{\infty }}=\;12.5$, and $R_{\infty }=$ 19,900 Ω/$\frac{\tau_{f}}{\tau_{\infty }}=\;1.25$, while $C_{\infty }$ is constant. Although the time constants have been kept equal to the ones shown in Figure 6A, adjusting their values by modifying $R_{\infty }$ has a stronger impact than adjusting them by changing $C_{\infty }$. This can be rationalized since, as explained above, the ($R_{\infty }C_{\infty }$) in parallel present their own semi-circle, the diameter of which is always dependent on the resistance (similarly to $R_{ct}$ in the faradaic semi-circle). The massive impact of $R_{\infty }$, especially in the case of $\frac{\tau_{f}}{\tau_{\infty }}=\;1.25$, conclusively proves that the time constant itself does not provide the full picture since the individual parameters contributing to it define the information depicted by the Nyquist plot.

An indicative example of the dielectric nature of a polymeric coating is given in [32]. This study used the impedance analysis of poly(dopamine)-coated electrodes to shed light on the mechanisms driving the interaction of proteins (bovine serum albumin (BSA) was used as a model) with poly(dopamine) in the presence of nucleophilic (Tris) and non-nucleophilic (phosphate) buffers. In this case, no external electroactive species were used in solution. Interestingly, when the Tris buffer is used both during dopamine polymerization and as a medium for protein adsorption, the Nyquist plot obtained closely matches the one depicted in Figure 6B for $\frac{\tau_{f}}{\tau_{\infty }}=\;12.5$. In this case, the authors attribute the low resistance, and thus high electron transfer, to the covalent binding of Tris to the poly(dopamine) coating that prevents BSA adsorption, and/or causes the incomplete polymerization of poly(dopamine). This conclusion is based on the relatively small $R_{ct}$ of the system, compared to the overall capacitance (in this case the $C_{\infty }$ is modeled by a CPE). Note that in this case, the charge transfer is not attributed to a redox reaction in solution, but to the redox processes (quinone to semi-quinone) inherent to the poly(dopamine) coating. While the presence of Tris, either during polymerization or as a medium for BSA adsorption, strongly protects from protein adsorption, the use of phosphate buffer promotes BSA adsorption. The absence of a competitive nucleophile favors BSA binding to poly(dopamine), which is supported by the significant increase in $R_{ct}$ upon BSA incubation (a behavior similar to the cases illustrated in Figure 2B). More examples regarding the influence of dielectric capacitance in EIS can be found here [33,34].

#### 3.2.2. Geometric Capacitance ($C_{g}$)

The geometric capacitance ($C_{g}$) is the capacitance formed between the working and counter electrodes, which is subject to any dielectric effects of the electrolyte. $C_{g}$ is given by Equation (17), where in this case ε is the relative permittivity (dielectric constant) of the electrolyte solution, and δ is the characteristic distance between the two electrodes. Additionally, Equation (19) also applies here, especially for measurements conducted in real-world samples with complex matrices. As an example, blood is a colloid with dielectric elements such as proteins, whose presence can reduce the conductivity of the measuring solution and increase the probability of $f_{c}$ emerging within the measurement range. However, unlike dielectric capacitance, which can often significantly impact EIS measurements, especially at high frequencies, geometric capacitance is rarely observed in real measurements.

Indeed, in most applications, $C_{g}$ can be considered negligible (typically showing very small values below 10^−11^ F [30]). Nonetheless, in some cases $C_{g}$ can cause significant interference within the high frequency range of the spectrum, affecting the determination of both $R_{\Omega }$ and $C_{dl}$.

Like $C_{\infty }$, $C_{g}$ also has its own time constant with $R_{\Omega }$, as per Equation (18). That means that all the examples illustrated in Figure 6 for $C_{\infty }$ are also valid for $C_{g}$ (albeit less often). A noteworthy case considering the effect of $C_{g}$ in a system consisting of multiple film–electrode interfaces is the impedimetric humidity sensor proposed by Trachioti et al. [35]. In this work, a 3-electrode screen-printed electrochemical cell was coated with a vanadium pentoxide xerogel (VPX). In this case, the VPX coating covered all three electrodes and their interfaces in the absence of any other electrolytic source. These EIS measurements were in the complete absence of electroactive species. That means that in low-humidity conditions, $R_{\Omega }$ was extremely high, exhibiting Nyquist plots similar to the one depicted in Figure 6B ($\frac{\tau_{f}}{\tau_{\infty }}=\;1.25$). Consequently, while humidity increased, $H_{2}O\;$ molecules were gradually intercalated into the VPX layered structure (leading to the formation of $V_{2}O_{5}\cdot H_{2}O$), increasing the ionic conductivity of the medium, and thus reducing $R_{\Omega }$, reaching impedimetric profiles similar to those for $\frac{\tau_{f}}{\tau_{\infty }}=\;12.5$. This application is one of the rare examples of the exploitation of the $C_{g}$ and $R_{\Omega }$ for an impedimetric sensor (obviously, excluding capacitance sensors where the impedance is measured at a single frequency).

#### 3.2.3. Pseudocapacitance ($C_{ps}$)

As mentioned above, dielectric electrode modifiers such as polymers can increase the dielectric capacitance of the electrode, causing a visible interference in the EI spectrum. However, certain conductive polymers, such as poly(aniline) (PANI), poly(pyrrole) (PPy), and poly(3,4-ethylenedioxythiophene) (PEDOT), can introduce a different type of capacitance into the electrochemical system, induced by inherent charge transfer processes [36]. The fact that this capacitive current (and hence the resulting capacitance) emerges as a response to a faradaic process necessitates the use of the “pseudo” prefix to avoid confusion with regular capacitance [37].

“Pseudocapacitance” is, in its essence, faradaic and stems from the ability of conductive polymers to be oxidatively doped (p-doping) and reductively doped (n-doping). When a conductive polymer is oxidatively p-doped (usually at a sufficiently anodic potential), it loses one or more electrons, creating an electron deficiency (“hole”) in its structure, causing the holes to become the prevalent charge carriers. At this stage, the polymer becomes positively charged, attracting negatively charged electrolyte ions to maintain electroneutrality [38]. The ions are inserted into the polymer’s structure, elevating its redox state and increasing the electrochemical system’s capacitance. Admittedly, p-doping is more effective in conductive polymers in terms of capacitance increase, especially in energy storage applications. However, in sensing applications, the presence of an inherent faradaic reaction of the electrode modifier, followed by a subsequent capacitance increase, can interfere with the sensing process. Alternatively, these “pseudocapacitive” processes can be utilized as sensing mechanisms, provided that a measurable difference in the faradaic current and/or capacitance (e.g., upon binding of a biomolecule) can be detected. Added to conductive polymers, other “pseudocapacitive” materials can be used as electrode modifiers in sensing applications, such as metal oxides (e.g., RuO_2_, IrO_x_, MnO_2_, etc.; in those cases, both n-doping and p-doping might give the pseudocapacitive impetus) [36]. Some of those oxides (e.g., IrO_x_) have been commonly used in potentiometric sensors, particularly as pH sensing probes [39,40].

**Figure 7:**
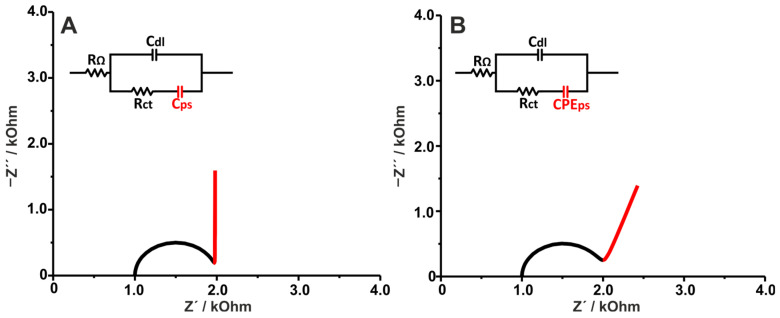
Simulated Nyquist plots showing (**A**) the effect of an ideal pseudocapacitive coating, $R_{\Omega }$ = 1000 Ω, $C_{dl}$ = 10^−5^ F, $R_{ct}$ = 1000 Ω, and $C_{ps}$ = 10^−3^ F; and (**B**) the effect of a non-ideal pseudocapacitive coating, $R_{\Omega }$ = 1000 Ω, $C_{dl}$ = 10^−5^ F, $R_{ct}$ = 1000 Ω, $Y_{dl}$ = 10^−3^ F s^n−1^, and n  = 0.8.

Figure 7 shows the simulated Nyquist plots of a pseudocapacitive electrode coated with pseudocapacitive materials. The effect of $C_{ps}$ emerges at lower frequencies, after the semi-circle attributed to the faradaic reaction of the pseudocapacitor. This is in line with the kinetics of such systems, since, as mentioned above, the faradaic reaction occurs first (is kinetically faster), thereby inducing an increase in capacitance. The difference between (A) and (B) is that in (A) the pseudocapacitance comes from an ideal capacitor element (where the reactance of the pseudocapacitor is parallel to the −Z” axis), while in (B) a CPE is used instead to denote the frequency dispersion in the pseudocapacitance. Both cases are common, and the outcome is usually dependent on the coherence of the coating and the presence of pores or defects.

Two important cases have to be considered: (1) if the pseudocapacitance is high enough (as shown in Figure 7, limited information on the mass transfer can be extracted (assuming there is mass transfer of electroactive species), and (2) if the faradaic time constant (see Equation (3)) of the electrode’s electroactive species is much larger than that of the electroactive species present in solution (e.g., in a (bio)sensing assay where redox species in solution are commonly used), then no reliable data can be extracted. Obviously, this also depends on the formal potential of the redox species and its relative proximity to the doping region of the pseudocapacitor.

The pseudocapacitive behavior shown by a conductive polymer-coated electrode is discussed in the study reported by Medhi et al., where the stepwise modification and antigen recognition of a capacitive immunosensor were electrochemically characterized [41]. More specifically, three electrodes, coated with PEDOT-PSS, graphene oxide (GO)/PEDOT-PSS, and gold nanoparticles (AuNPs)/GO/PEDOT-PSS, were characterized via EIS to confirm the optimal electrode composition after glutaraldehyde crosslinking, antibody immobilization, and antigen interaction. First, an obvious increase in $R_{ct}$ was observed after each biosensor modification step, as expected for the reasons discussed in Section 3.1.1. Note that no external redox molecule was used, so $R_{ct}$ refers to the charge transfer resistance of the PEDOT:PSS. Furthermore, according to the authors, the dielectric capacitance significantly increased after glutaraldehyde treatment, likely due to the introduction of reactive di-aldehydic groups over the electrode. Then, the dielectric capacitance slightly decreased after antibody immobilization, an effect attributed to the additional dielectric layer of the neutral antibody introduced between the electrode–electrolyte interface. Similarly, the dielectric capacitance also decreased after antigen binding, suggesting the formation of a closely bound antibody–antigen complex with associated conformational changes in the proteins. Indeed, the antigen–antibody interactions are highly specific, with the resulting complex forming a compact dielectric layer. Regarding the pseudocapacitance itself (which was modeled by a CPE, similar to Figure 7B), it is interesting to note that the overall value remains virtually unaffected after antibody immobilization and antigen interaction, except by a decrease in n on the functionalized electrode suggesting inhomogeneity in the space–charge layer between PEDOT, PSS grains and GO/Au, due to the adsorption of both the antibody and antigen. More examples regarding the influence of pseudocapacitance on EIS are provided here [42,43].

## 4. Discussion

This review discusses selected simulated examples of common (bio)sensing scenarios where the standard Randles circuit cannot fully describe the involved electrochemical phenomena. Certain modifications of the Randles circuit are suggested to address cases where different mass transfer regimes or capacitances are involved.

First, this review examines the impact of the semi-infinite mass transfer regime on the data obtained from the EI spectra. Simulations show that the parameter causing the most significant increase in the mass transfer/faradaic time constant ratio, increasing $R_{ct}$ impacts heavily the Nyquist plots through the disappearance of the mass transfer part of the plot, while the same ratio increase achieved via $C_{dl}$ does not impart a similar significant effect. Thus, the ratio of the two time constants is not the single-handedly defining factor to attain the best resolution between the kinetic and mass transfer parts of the plot. Next, the effect of (hemi)spherical mass transfer was examined. In this case, active and inactive parts of the electrode surface affect the relationship with the diffusion layer. Hemispherical mass transfer takes place when the diffusion layer is larger than the mean radius of the active centers but smaller than the mean radius of the inactive centers. It is further shown that large active radii (simulating either the radii of ultramicroelectrodes or the active areas of heterogeneous electrode surfaces) impact the mass transfer leading to linear diffusion, due to the planar behavior of the electrode, while small active radii result in hemispherical mass transfer (assigned by low-frequency curvature).

This review also discusses the different capacitance regimes influencing the Randles circuit. The effect of an electrode coating on the dielectric capacitance was investigated through the analysis of simulated Nyquist plots corresponding to different ratios of faradaic to dielectric time constants, obtained by varying the bulk resistance or dielectric capacitance. Simulations indicate that the dielectric time constant significantly influences the outcome of the Nyquist plot. Moreover, adjusting the time constant ratio via changes in the bulk resistance strongly impacts the dielectric capacitance. The effect of the geometric capacitance (the capacitance induced between the working and counter electrodes) has a similar effect on the Nyquist plots. Finally, the impact of pseudocapacitance (capacitance induced by faradaic reactions) on the low-frequency part of the impedance plots was illustrated. In this case, pseudocapacitance might be modeled either by an ideal capacitor element or by a CPE (used instead to denote the frequency dispersion). When pseudocapacitance is high enough, the amount of information extracted on the mass transfer is limited. Moreover, if the faradaic time constant of the coating’s electroactive species is much larger than that of the electroactive species present in solution, then extracting analytically important data will be questionable.

## 5. Conclusions

As intended, the simulated signals discussed in this work correlate well with real-life (bio)sensing examples and thus can be used to critically analyze the impact of choosing the right equivalent circuit. It is within the goal of this work to instill in the reader the view that not all impedimetric data can be mathematically fitted, and/or contain meaningful physicochemical characteristics of each system. In such cases, knowledge of the electrochemical system plays a pivotal role in identifying the most appropriate equivalent circuit, thereby increasing the accuracy and reliability of scientific results.

## Data Availability

The data that support the findings of this study are available from the corresponding author, A.L., upon reasonable request.
